# TRIM28 facilitates type I interferon activation by targeting TBK1

**DOI:** 10.3389/fimmu.2024.1279920

**Published:** 2024-03-01

**Authors:** Fang Hua, Tim Nass, Kislay Parvatiyar

**Affiliations:** Department of Microbiology and Immunology, Tulane University School of Medicine, New Orleans, LA, United States

**Keywords:** TRIM28, tripartite motif containing 28, TBK1, type I interferon (IFN), pattern recognition receptor (PRR)

## Abstract

Type I interferons play a fundamental role in innate host defense against viral infections by eliciting the induction of an antiviral gene program that serves to inhibit viral replication. Activation of type I interferon is regulated by the IRF3 transcription factor, which undergoes phosphorylation-dependent activation by the upstream kinase, TBK1, during viral infection. However, the mechanisms by which TBK1 achieves activation to support signaling to IRF3 remain incompletely understood. Here we identified the E3 ubiquitin ligase, tripartite motif containing 28 (TRIM28), as a positive regulator of type I interferon activation by facilitating TBK1 signaling. Genetic deletion of TRIM28 via CRISPR-Cas9 editing resulted in impaired type I interferon activation upon both RNA and DNA virus challenge, corresponding with increased susceptibility to virus infections in TRIM28 knockout cells. Mechanistically, TRIM28 interacted with TBK1 and mediated the assembly of K63-linked ubiquitin chains onto TBK1, a post-translational modification shown to augment TBK1 signal transmission events. TRIM28 knockout cells further displayed defective TBK1 phosphorylation and complex assembly with IRF3, resulting in impaired IRF3 phosphorylation. Altogether, our data demonstrate TBK1 to be a novel substrate for TRIM28 and identify TRIM28 as an essential regulatory factor in controlling innate antiviral immune responses.

## Introduction

The innate arm of the immune system provides a first line of defense against invading pathogens. In the context of viral infections, the induction of the type I interferon (IFN-I) cytokines (IFNα/β) is critical in rapidly coordinating an antiviral cellular state and the initiation of a secondary, T-cell-mediated adaptive immune response (1, 2). IFN-I activation is regulated at the level of transcription, primarily by the interferon regulatory factor 3 (IRF3) transcription factor, which undergoes phosphorylation-dependent activation via TANK binding kinase 1 (TBK1) (3, 4). Activation of the TBK1-IRF3 axis is governed via select germ-line encoded pattern recognition receptors (PRRs) that detect viral genomes in the cytosol of the infected cell (1, 5). The genomes of RNA viruses are typically detected by the retinoic acid inducible gene I (RIG-I) or melanoma differentiation associated protein 5 (MDA5) PRRs which activate TBK1-IRF3 via the mitochondrial antiviral signaling (MAVS) adaptor (RNA pathway). Alternatively, the genomes of DNA viruses are detected by cyclic guanosine adenosine monophosphate synthase (cGAS), DEAD-box helicase 41 (DDX41), or interferon gamma inducible protein 16 (IFI16). These cytosolic DNA sensing PRRs signal to TBK1-IRF3 via a separate adaptor, a stimulator of interferon genes (STING) (DNA pathway) (2, 5–7). While much attention has focused on delineating the mechanisms by which the RNA vs DNA sensing pathways are regulated, it remains incompletely understood how these disparate PRR pathways confer downstream signaling to TBK1 to achieve IRF3 activation and IFN-I induction to mediate antiviral host defenses.

Post-translational modifications, particularly protein ubiquitination, have been implicated in regulating a wide variety of cellular processes, including innate antiviral host defense pathways (8, 9). Ubiquitin is composed of 76 amino acids, which includes seven lysine residues. While the conjugation of a single ubiquitin (monoubiquitination) onto a target substrate often plays a role in regulating protein transport/localization or DNA repair, the addition of sequential ubiquitin moieties that result in the assembly of a ubiquitin polymeric chain (polyubiquitination) on target protein substrates fosters various outcomes that are dependent on the format by which the ubiquitin oligomers are linked. The best characterized types of polyubiquitin chains are those that are interlinked via lysine residues at position 48 (K48) or position 63 (K63). K48-linked polyubiquitination of proteins typically instigates proteasomal targeting and subsequent protein degradation. Alternatively, K63-linked polyubiquitination confers non-degradative outcomes, which support downstream signal transduction events (10, 11). For example, K63-linked ubiquitination of the RNA pathway PRR RIG-I, mediated by the E3 ubiquitin ligase ring finger protein 135 (RNF135, aka Riplet), results in RIG-I oligomerization and downstream signal amplification (12, 13). Likewise, K63-linked ubiquitination of the RNA pathway adaptor MAVS by the tripartite motif 31 (TRIM31) E3 ligase was shown to promote MAVS aggregation and activation (14). In the DNA pathway, the STING adaptor is subjected to K63-linked ubiquitination by TRIM56, TRIM32, and mitochondrial E3 ubiquitin protein ligase 1 (MUL1, aka RNF218), which promote STING signaling (14, 15). Downstream of the RNA or DNA pathway signaling adaptors, the kinase TBK1 has been observed by several groups to undergo K63-linked ubiquitination to facilitate its activation, however the E3 ubiquitin ligase(s) that control K63-linked ubiquitination of TBK1 in both the RNA and DNA pathway remain poorly defined (9, 14, 16, 17).

TRIM28 (aka KAP1) is an E3 ubiquitin ligase that has garnered significant attention in the area of cancer biology as its expression is linked with tumor progression (18–20). Additional data establish a role for TRIM28 in controlling endogenous retroviruses and transposable elements via mechanisms involving chromatin remodeling, while older studies suggest TRIM28 suppresses innate antiviral immune responses by targeting the IRF3-related transcription factors, IRF5/7 (21–27). Notably, the roles of certain E3 ubiquitin ligases in innate immunity have been met with controversy as initial conclusions were based upon experimental approaches that relied heavily on gain of function studies along with small interfering or short hairpin RNA silencing strategies that failed to recapitulate phenotypes observed in genetic knockout cells (28, 29). To minimize artifactual and potentially erroneous findings, we employed a loss of function approach using CRISPR-Cas9 gene editing tools to examine the role of TRIM28 in innate antiviral signaling and host defense. Our data identifies TRIM28 as a positive regulator of IFN-I activation and cellular antiviral responses downstream of cytosolic RNA and DNA PRRs. Further biochemical analysis indicates TRIM28 interacts with TBK1 and mediates TBK1 K63-linked ubiquitination and association with the IRF3 transcription factor to drive IFN-I activation.

## Results

### TRIM28 is a positive regulator of IFN-I in the cytosolic RNA and DNA sensing pathway

**Figure 1:**
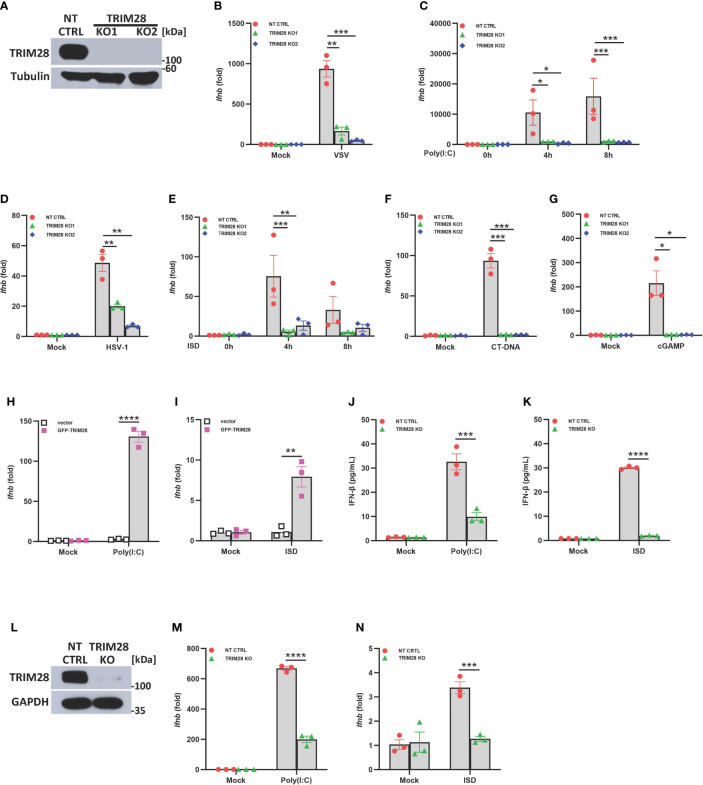
TRIM28 is a positive regulator of IFN-I activation in the cytosolic RNA and DNA sensing PRR pathways. **(A)** Immunoblot analysis of TRIM28 expression in single guide (sg) RNA non-targeting control (NT CTRL) and sgRNA targeting TRIM28 (TRIM28 knockout clone 1, KO1; and knockout clone 2, KO2) murine embryonic fibroblasts (MEFs). Results are representative of three independent experiments. **(B–G)** qPCR analysis of IFN-β mRNA induction in NT CTRL and TRIM28 KO MEFs **(B)** infected with vesicular stomatitis virus (VSV) (MOI 0.1, 24 h), **(C)** transfected with poly I:C (2.5 µg/mL), **(D)** infected with herpes simplex virus 1 (HSV-1) (MOI 1.0, 12 h), **(E)** transfected with immunostimulatory DNA (ISD) (2 µg/mL), **(F)** transfected with calf-thymus DNA (CT-DNA) (2 µg/mL, 8 h), or **(G)** transfected with 2’3’-cyclic GMP-AMP (cGAMP) (2 µg/mL, 8 h) as indicated. **(H-I)** qPCR analysis of IFN-β mRNA induction in TRIM28 KO MEFs reconstituted with empty vector or GFP-TRIM28 plasmid for 24 h and subsequently transfected with **(H)** poly I:C (5 µg/mL, 4 h) or **(I)** ISD (10 µg/mL, 4 h). **(J, K)** ELISA for IFN-β production in NT CTRL and TRIM28 KO MEFs transfected with **(J)** poly I:C (4 µg/mL, 24 h) or **(K)** ISD (20 µg/mL, 24 h). **(L)** Immunoblot analysis of TRIM28 expression in NT CTRL and TRIM28 KO U937 cells. **(M, N)** qPCR analysis of IFN-β mRNA induction in NT CTRL and TRIM28 KO U937 cells transfected with **(M)** poly I:C (6 µg/mL, 16 h) or **(N)** ISD (3 µg/mL, 16 h). Data represent means ± SEM of three independent experiments. Statistical significance was determined using ANOVA or student’s t-test (****P<0.0001, ***P<0.001, **P<0.01, and *P<0.05).

To refine our understanding of the role of TRIM28 in innate antiviral immunity, we first utilized CRISPR-Cas9 gene editing technology to generate TRIM28 knockout (KO) murine embryonic fibroblast (MEF) cell lines (**Figure 1A**). To determine if TRIM28 played a role in regulating IFN-I activation in cytosolic nucleic acid sensing PRR pathways, non-targeting control (NT CTRL) and TRIM28 KO MEF cells were infected with the RNA virus and vesicular stomatitis virus (VSV) or transfected with the double-stranded RNA mimetic poly (I:C), an RNA PRR pathway inducer. While non-targeting control cells displayed robust activation of IFN-I, TRIM28 KO cells were significantly defective in inducing IFN-I (**Figures 1B, C**). Non-targeting control and TRIM28 KO MEF cells were also infected with the DNA virus and herpes simplex virus 1 (HSV-1) or transfected with DNA sensing PRR pathway inducers immunostimulatory DNA (ISD), calf thymus DNA (CT-DNA), and cyclic-GMP-AMP (cGAMP). Similar to our observations in the RNA pathway, cells lacking TRIM28 were impaired in IFN-I activation compared to non-targeting control cells in the DNA pathway (**Figures 1D–G**). To determine whether the defective IFN-I response exhibited during cytosolic nucleic acid stimulation was indeed imparted by the loss of TRIM28, MEF cells deficient for TRIM28 were reconstituted with either empty vector or a plasmid encoding TRIM28 followed by RNA or DNA pathway activation. While cells lacking TRIM28 expectedly failed to elicit IFN-I, TRIM28 KO cells that were reconstituted with TRIM28 showcased significant increases in IFN-I induction in both the RNA and DNA pathway, rescuing the impaired IFN-I phenotype observed in the TRIM28 KO cells (**Figures 1H, I**). We additionally assessed the role of TRIM28 in the downstream production of the IFN-I cytokine, IFNβ. In agreement with our data that TRIM28 necessitates IFN-I induction, we found TRIM28 was also required for IFN-I production as MEF cells lacking TRIM28 presented markedly reduced levels of IFNβ secretion compared to non-targeting control cells upon RNA or DNA pathway stimulation (**Figures 1J, K**). To further confirm a critical role for TRIM28 as a positive regulator of IFN-I, we deleted TRIM28 in the human U937 monocyte cell line via CRISPR-Cas9 gene editing (**Figure 1L**). Similar to TRIM28 KO MEF cells, U937 cells lacking TRIM28 also displayed impaired IFN-I responses compared to control cells during cytosolic nucleic acid stimulation (**Figures 1M, N**).

**Figure 2:**
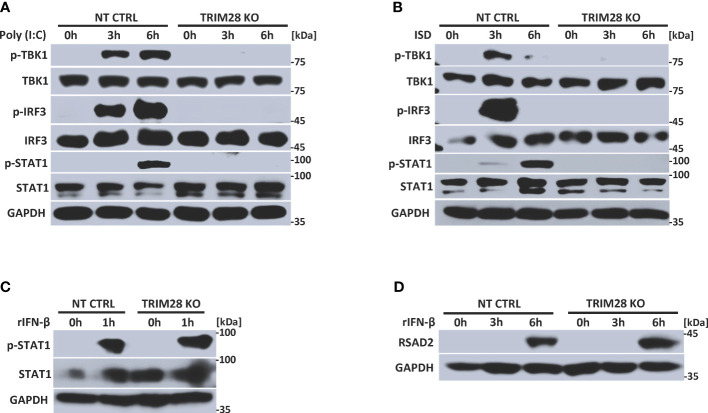
TRIM28 is required for RNA and DNA pathway signaling to IRF3. **(A, B)** Immunoblot analysis of TBK1 (S172), IRF3 (S396), and STAT1 (Y701) phosphorylation in NT CTRL and TRIM28 KO MEFs transfected with **(A)** poly I:C (2.5 µg/mL) or **(B)** ISD (2 µg/mL) for the indicated time points. **(C, D)** Immunoblot analysis of **(C)** STAT1 (Y701) phosphorylation and **(D)** RSAD2 expression in NT CTRL and TRIM28 KO MEFs treated with recombinant IFN-β (100 U/mL) for the indicated times. Results are representative of two independent experiments.

**Figure 3:**
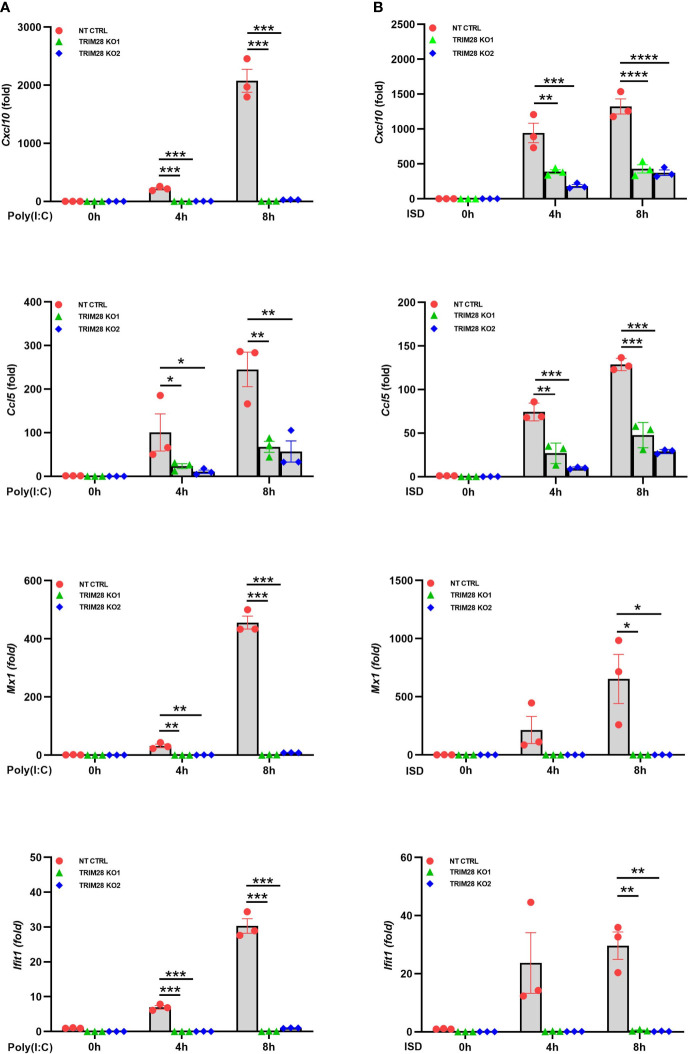
TRIM28 is essential for the downstream activation of interferon stimulated genes. **(A, B)** qPCR analysis of interferon stimulated genes IP-10 (Cxcl10), RANTES (Ccl5), Mx1, and Ifit1 in NT CTRL and TRIM28 KO MEFs transfected with **(A)** poly I:C (2.5 µg/mL) or **(B)** ISD (2 µg/mL) as indicated. Data represent means ± SEM of three independent experiments. Statistical significance was determined using ANOVA or student’s t-test (****P<0.0001, ***P<0.001, **P<0.01, and *P<0.05).

IFN-I induction in the RNA and DNA pathway is dependent upon phosphorylation of the IRF3 transcription factor, which is activated via the upstream kinase TBK1 upon engagement of cytosolic nucleic acid sensing PRRs (4, 5). Corresponding with the defective IFN-I activity in TRIM28 KO cells, MEF cells lacking TRIM28 additionally displayed impaired activation of TBK1 and IRF3 as their phosphorylation profiles were significantly reduced during RNA or DNA pathway stimulation compared to non-targeting control cells (**Figures 2A, B**). IFN-I operates in an autocrine and paracrine fashion to engage the IFNα/β receptor (IFNAR), resulting in the activation of a Janus kinase-signal transducer and activator of transcription (JAK-STAT) signaling pathway that orchestrates the induction of several hundred interferon stimulated genes (ISGs) that collectively foster an antiviral state (2). While non-targeting control cells displayed intact STAT1 phosphorylation, TRIM28 KO cells were highly defective for STAT1 activation in both the RNA and DNA pathway (**Figures 2A, B**). Alternatively, TRIM28 was specific in mediating STAT1 and ISG activation upon cytosolic nucleic acid PRR ligation and not upon downstream IFN-I stimulation as IFNAR signaling remained intact in TRIM28 KO cells which showed no marked defects in STAT1 phosphorylation (**Figure 2C**) or induction of the ISG, RSAD2 (**Figure 2D**) in comparison to IFN-I treated non-targeting control cells. However, consistent with its key role in facilitating STAT1 activation in the cytosolic nucleic acid sensing PRR pathways, induction of the ISGs IP10 (aka CXCL10), RANTES (aka CCL5), MX1, and IFIT1 was severely limited in cells lacking TRIM28 when compared to control cells (**Figures 3A, B**). Taken together, these results suggest TRIM28 plays a critical role in eliciting IFN-I activation in both cytosolic RNA and DNA sensing pathways.

### TRIM28 interacts with TBK1

**Figure 4:**
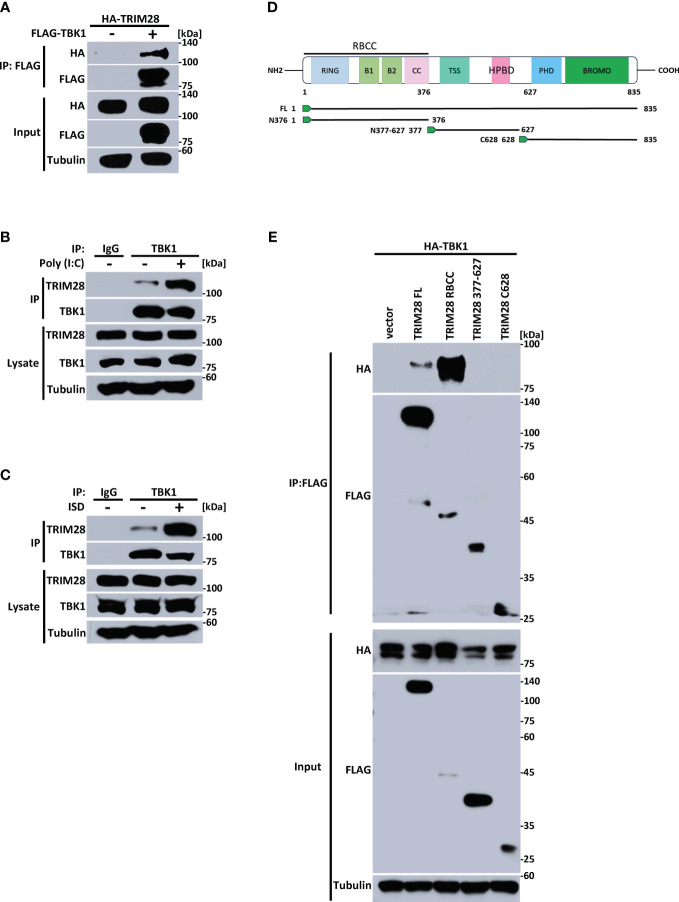
TRIM28 interacts with TBK1. **(A)** Co-immunoprecipitation and immunoblot of HA-TRIM28 and FLAG TBK1 co-transfected in HEK 293T cells. **(B, C)** Immunoblot analysis of TRIM28-TBK1 interactions in MEF cells transfected with **(B)** poly I:C (2.5 µg/mL) or **(C)** ISD (2 µg/mL) for 3 h followed by control IgG or TBK1 immunoprecipitation as indicated. **(D)** Schematic representation of TRIM28 domains and deletion constructs generated. **(E)** Co-immunoprecipitation and immunoblot of FLAG-TRIM28 full length (FL), amino-terminal (RBCC), central (377-627), or carboxyl terminal 628-835 (C628) with HA-TBK1 co-transfected in HEK 293T cells. Results are representative of two independent experiments.

The cytosolic accumulation of RNA vs DNA species triggers the activation of differing PRRs and separate signaling adaptors that converge at TBK1 to activate IRF3 and instigate IFN-I induction (2, 5, 30). As our data points to an essential role for TRIM28 in controlling IFN-I activation in both the RNA and the DNA pathway, we reasoned TRIM28 to operate via TBK1, a downstream signaling protein common to both pathways. Indeed, ectopic expression of TRIM28 with TBK1 in HEK 293T cells showed that TRIM28 interacted with TBK1 (**Figure 4A**). At the endogenous level, TRIM28 is also associated with TBK1, which was further enhanced upon RNA or DNA pathway stimulation (**Figures 4B, C**). TRIM28 is composed of an amino terminal RING, B-box, coiled-coil (RBCC) motif, a central TRIM specific sequence (TSS) and Heterochromatin Protein 1 binding domain (HPBD), and a carboxyl terminus that contains a plant homeodomain (PHD) finger and a bromodomain (BROMO) (**Figure 4D**). To identify the domain(s) of TRIM28 that are responsible for its interaction with TBK1, we generated TRIM28 plasmid constructs expressing amino terminal, central, or carboxyl terminal domains. TBK1 co-immunoprecipitation analysis revealed that the amino terminal RBCC domain of TRIM28 was required for TBK1 interaction (**Figure 4E**). Together, these results suggest TRIM28 functions in activating IFN-I in the RNA and DNA sensing pathways by targeting the shared signaling component, TBK1.

### TRIM28 facilitates TBK1-IRF3 complex formation

**Figure 5:**
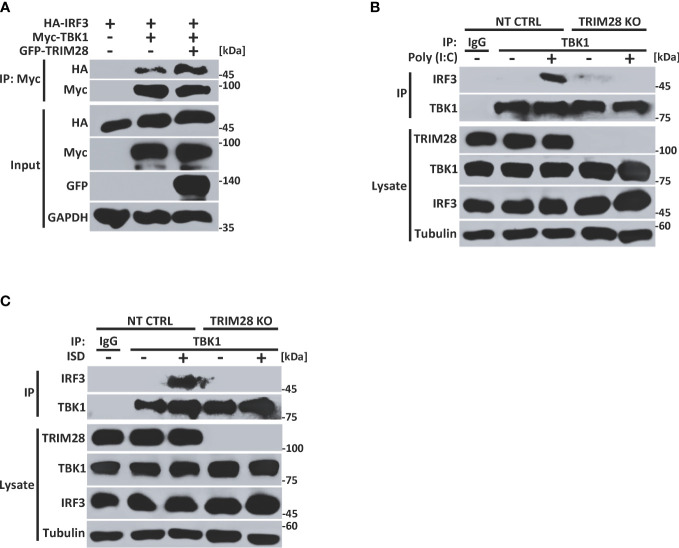
TRIM28 necessitates TBK1-IRF3 interactions. **(A)** Co-immunoprecipitation and immunoblot analysis of Myc-TBK1 and HA-IRF3 in the presence or absence of GFP-TRIM28 co-transfected in HEK 293T cells. **(B, C)** Immunoblot analysis of TBK1-IRF3 interactions in NT CTRL and TRIM28 KO MEF cells transfected with **(B)** poly I:C (2.5 µg/mL) or **(C)** ISD (2 µg/mL) for 3 h followed by control IgG or TBK1 immunoprecipitation as indicated. Results are representative of two independent experiments.

Cells lacking TRIM28 display defective TBK1 phosphorylation and subsequent downstream signaling events (e.g., IRF3 phosphorylation and IFN-I activation) in the cytosolic RNA and DNA sensing pathways (**Figure 2**). As our data revealed TRIM28 to interact with TBK1, we next wanted to examine how TRIM28 controlled TBK1 signaling events. TRIM28 expression in HEK 293T cells resulted in enhanced interactions between TBK1 and IRF3 (**Figure 5A**). Furthermore, while non-targeting control MEF cells formed TBK1-IRF3 complexes upon RNA or DNA pathway stimulations, TRIM28 KO MEF cells were deficient in establishing TBK1-IRF3 interactions (**Figures 5B, C**). These data suggest TRIM28 plays a role in mediating IFN-I activation by interacting with TBK1 and necessitating its association with IRF3.

### TRIM28 mediates K63-linked ubiquitination of TBK1

**Figure 6:**
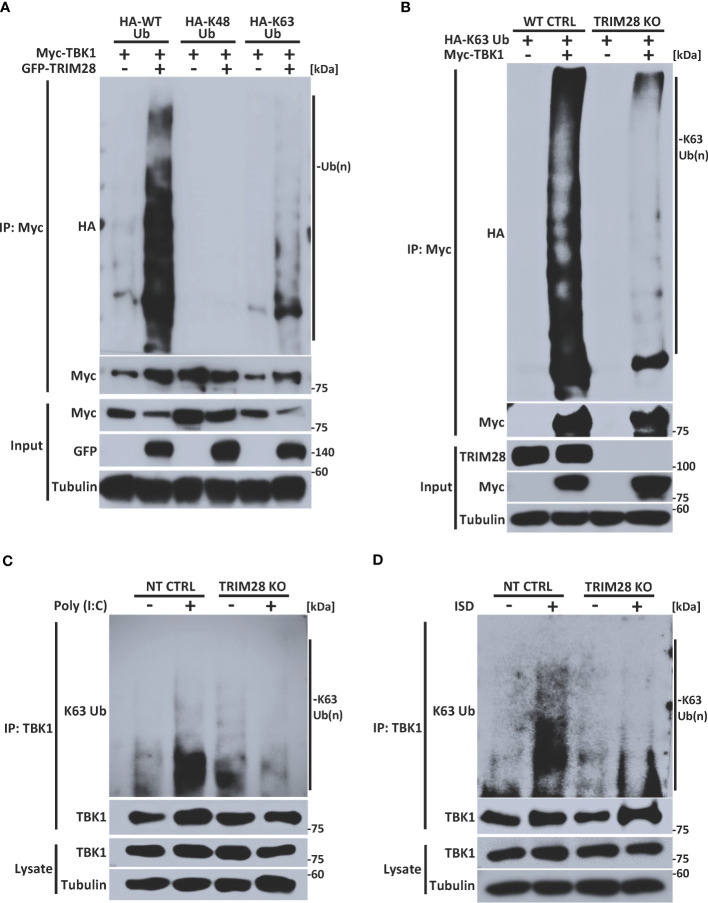
TRIM28 facilitates TBK1 K63-linked ubiquitination. **(A)** Immunoprecipitation and immunoblot analysis of HA-WT (total), K48, or K63 -linked ubiquitin chains covalently attached to Myc-TBK1 in the absence or presence of GFP-TRIM28 in HEK 293T cells. **(B)** Immunoprecipitation and immunoblot of HA-K63-linked ubiquitin chains covalently attached to Myc-TBK1 in WT or TRIM28 KO HEK 293T cells. **(C, D)** Immunoblot analysis of K63-linked ubiquitin chains attached to TBK1 immunoprecipitated from NT CTRL or TRIM28 KO MEFs transfected with **(C)** poly (I:C) (3 µg/mL) or **(D)** ISD (4 µg/mL) for 2 h. Results are representative of two independent experiments.

Many members of the TRIM family of proteins, including TRIM28, contain an amino terminal RING domain which confers E3 ubiquitin ligase function to mediate the assembly of K48 or K63-linked polyubiquitin chains onto a target substrate (14, 28, 31, 32). Previous studies have reported that TBK1 is subjected to non-degradative K63-linked ubiquitination and that this post-translational modification plays a key role in driving TBK1-dependent signaling to IRF3 and activation of IFN-I (14, 16, 17). To determine whether TRIM28 could modulate TBK1 ubiquitination, TBK1 and TRIM28 were co-expressed with plasmids encoding WT ubiquitin or ubiquitin mutants harboring arginine substitutions at all lysine residues except at positions 48 or 63 (e.g., only capable of K48 or K63-linked ubiquitin assembly, respectively). Ectopic expression of TRIM28 resulted in elevated TBK1 ubiquitination, and specific ubiquitin linkage analysis indicated an increase in K63-linked -but not K48-linked- ubiquitination of TBK1 in the presence of TRIM28 (**Figure 6A**). To further corroborate the role of TRIM28 in mediating K63-linked ubiquitination of TBK1, we generated TRIM28 KO HEK 293T cells via CRISPR-Cas9 gene editing and examined the K63-linked ubiquitination profile of TBK1. As expected, TBK1 was robustly modified with K63-linked polyubiquitin in control cells, however the assembly of K63-linked ubiquitin chains onto TBK1 was significantly impaired in cells lacking TRIM28 (**Figure 6B**). Consistently, both RNA and DNA pathway stimulation triggered the conjugation of endogenous K63-linked ubiquitin chains onto TBK1, which was largely absent in TRIM28 KO MEF cells (**Figures 6C, D**).

**Figure 7:**
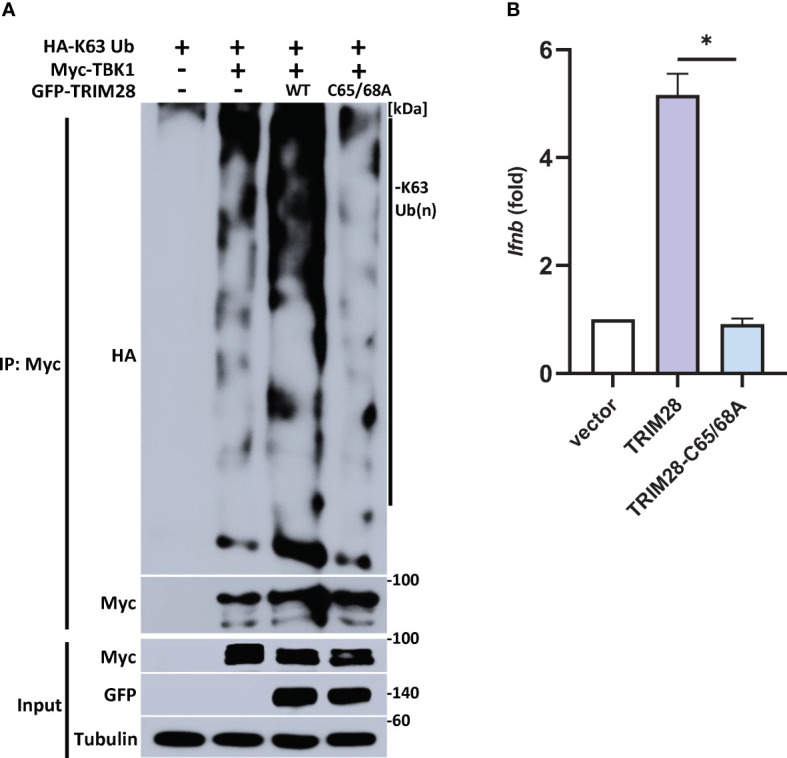
TRIM28 requires E3 ligase function to mediate TBK1 K63-linked ubiquitination and IFN-I activation. **(A)** Immunoprecipitation and immunoblot analysis of HA-K63-linked ubiquitin chains covalently attached to Myc-TBK1 in TRIM28 KO HEK 293T cells reconstituted with WT or catalytically inactive (C65/68A) GFP-TRIM28. Results are representative of two independent experiments. **(B)** qPCR analysis of IFN-β mRNA induction in TRIM28 KO MEF cells reconstituted with empty vector, WT TRIM28, or catalytically inactive TRIM28. Data represent means ± SEM of two independent experiments. Statistical significance was determined using ANOVA or student’s t-test (****P<0.0001, ***P<0.001, **P<0.01, and *P<0.05).

TRIM28 E3 ubiquitin ligase function has been reported to require two cysteine residues (C65/C68) located within its RING domain (33–37). To further define a role for TRIM28 in catalyzing TBK1 K63-linked ubiquitination, TRIM28 KO HEK 293T cells were reconstituted with either WT TRIM28 or an E3 ligase incompetent TRIM28 (TRIM28 C65A/C68A) in the presence of TBK1 and a K63-ubiquitin specific plasmid. While WT TRIM28 increased TBK1 K63-linked ubiquitination, the assembly of K63-linked ubiquitin chains on TBK1 was severely diminished when co-expressed with the E3 ligase mutant TRIM28 (**Figure 7A**). Accordingly, the E3 ubiquitin ligase function of TRIM28 was required to mediate IFN-I activation as TRIM28 KO MEF cells reconstituted with only WT TRIM28 but not the catalytically inactive TRIM28 mutant was competent to induce IFN-I (**Figure 7B**). Collectively, these findings suggest TRIM28 facilitates K63-linked ubiquitination of TBK1 to support its activation of IFN-I.

### TRIM28 is required for cellular antiviral host defense

**Figure 8:**
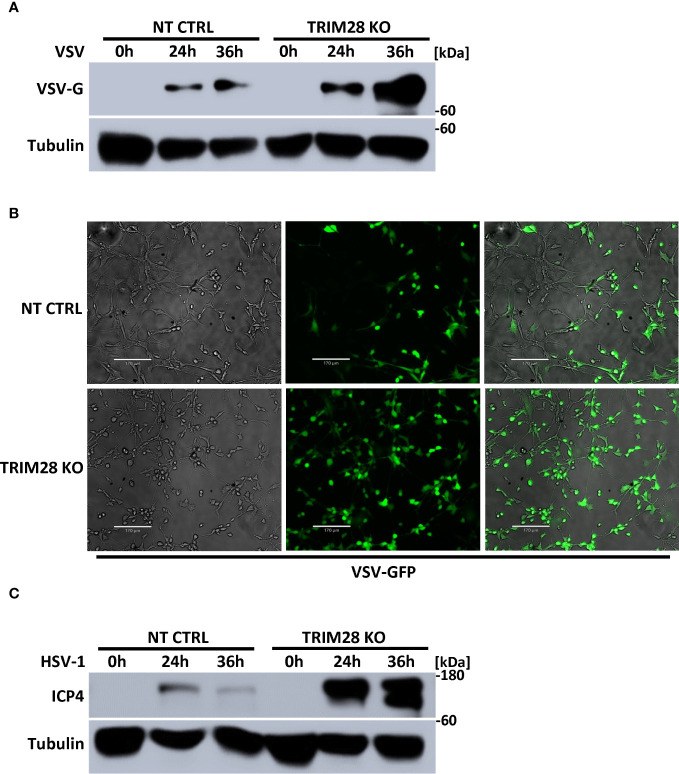
TRIM28 is necessary for the cellular antiviral response. **(A)** Immunoblot analysis for VSV encoded glycoprotein, G (VSV-G) in NT CTRL and TRIM28 KO MEF cells infected with VSV (MOI 0.1) for the indicated times. **(B)** Fluorescent imaging of NT CTRL and TRIM28 KO MEFs infected with VSV-GFP (MOI 0.1) for 16 h. Left to right: bright field, GFP, merged channels. Scale bar = 170 µm. **(C)** Immunoblot analysis of HSV-1 encoded immediate early transcription factor, ICP4 (HSV-1 ICP4) in NT CTRL and TRIM28 KO MEF cells infected with HSV-1 (MOI 1.0) for the indicated times. Results are representative of two independent experiments.

The induction of IFN-I is fundamental in eliciting innate antiviral host defense as it functions to inhibit viral replication and spread (1, 2). To determine the physiological role of TRIM28 in the context of viral infections, non-targeting control and TRIM28 KO MEF cells were infected with the RNA virus, VSV. Cells lacking TRIM28 displayed elevated levels of the VSV encoded glycoprotein G (**Figure 8A**), corresponding with increased susceptibility to viral infection compared to control cells (**Figure 8B**). Likewise, TRIM28 KO MEF cells infected with the DNA virus, HSV-1 failed to suppress viral replication as they exhibited greater levels of the HSV-1 encoded protein, ICP4 in comparison to control cells (**Figure 8C**). Together, these results reveal TRIM28 plays an essential role in controlling RNA and DNA viral replication.

## Discussion

**Figure 9:**
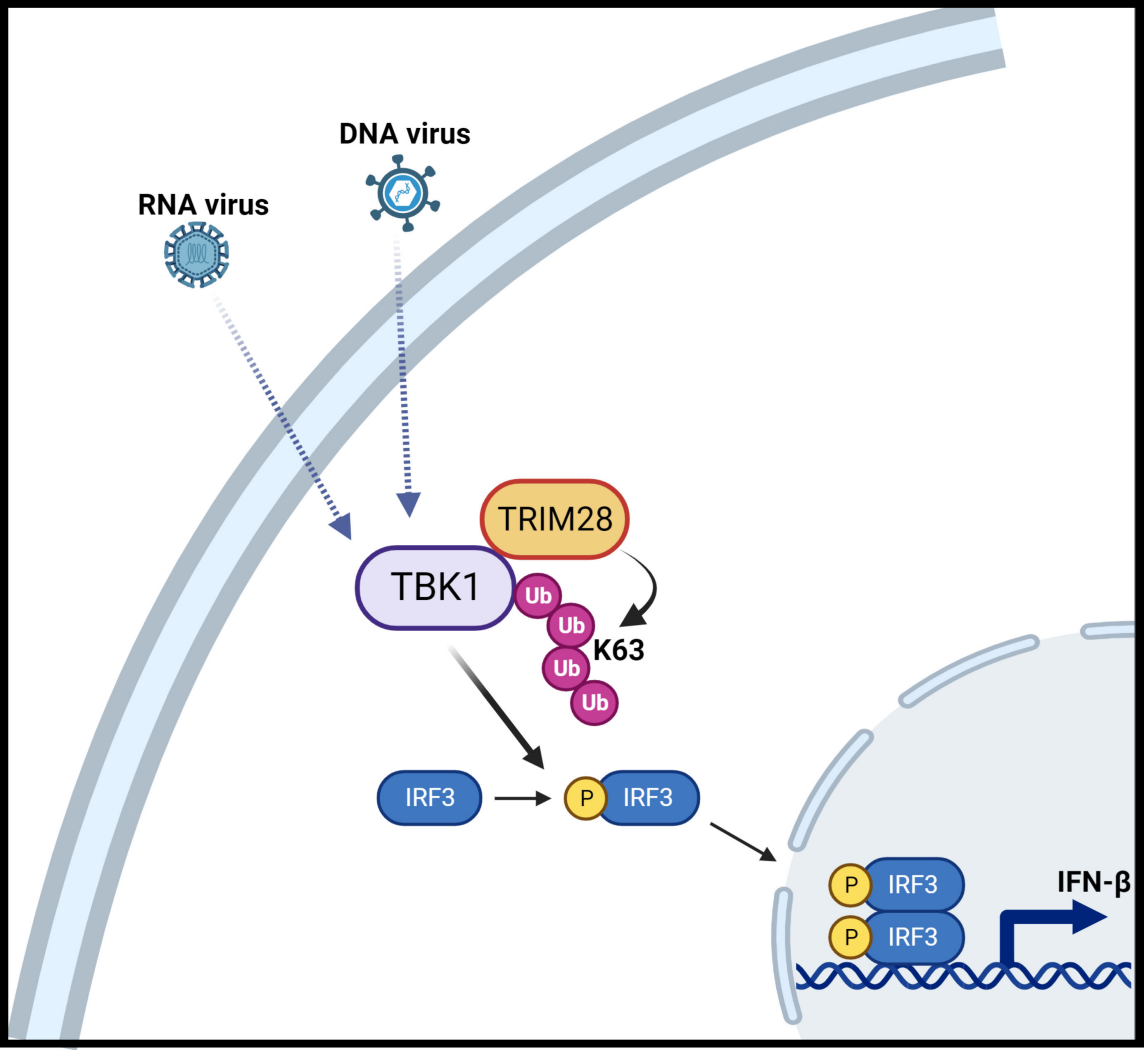
Schematic illustration of TRIM28 function in cytosolic nucleic acid PRR signaling. Detection of RNA or DNA virus genomes by distinct cytosolic pattern recognition receptors (PRRs) converge upon TBK1 which is associated with TRIM28. RNA or DNA PRR pathway engagement triggers TRIM28 mediated K63-linked ubiquitination of TBK1 which necessitates TBK1 activation and downstream signaling to IRF3 to elicit IFN-β induction. Figure created with BioRender.

The role of post-translational modifications, including the conjugation of non-degradative, K63-linked ubiquitin polymers onto key signaling proteins, has been demonstrated to support signal transduction events in a variety of innate immune pathways (9–11). Accordingly, several E3 ubiquitin ligases have been identified as regulators of innate signaling, controlling the activation of IFN-I downstream of nucleic acid sensing PRRs. Here, we provide evidence that the TRIM28 E3 ubiquitin ligase operates as a positive regulator of the host antiviral IFN-I response as TRIM28 KO cells display impaired IFN-I activation in both the RNA and DNA sensing PRR pathways (**Figure 9**). Consistent with these observations, we found TRIM28 KO MEF cells were defective in signaling to IRF3, resulting in impaired downstream activation of the JAK-STAT antiviral pathway to consequently yield attenuated viral restriction capabilities. In contrast, a recent study found TRIM28 inhibited IFN-I activation exclusively in the RNA pathway by targeting MAVS for K48-linked ubiquitination and subsequent degradation, while a previous report found TRIM28 suppressed IFN-I via a mechanism that targeted IRF7 for small ubiquitin-like modifier (sumo) ligation (sumoylation) (26, 38). New data reveal that monocytes genetically deleted for sumo ligases or sumoylation machinery displayed spontaneous activation of the IFN-I response but in a manner independent of IRF7 or IRF3 (39). As such, it is an intriguing possibility that TRIM28 may coordinate with various E2 -ubiquitin as well as -sumo conjugating enzymes to deliver polyubiquitin chains with differing linkages or sumo, respectively to modulate target protein function (40). Nevertheless, the disparities in our findings from the previous published works may likely be explained by the differing approaches employed to examine TRIM28 function. Notably, both of the above studies utilized experimental designs that relied on over-expression of genes encoded on plasmids in combination with RNA silencing techniques in cell lines that harbor incomplete RNA or DNA sensing PRR pathway components. Alternatively, we primarily utilized a CRISPR-Cas9 mediated gene knock out, loss of function approach to evaluate the innate antiviral role of TRIM28 in MEF cells, known to have intact RNA and DNA PRR signaling pathways. Interestingly, a recent report found viral infection could trigger TRIM28 S473 phosphorylation, through a protein kinase R (PKR) - p38 mitogen activated protein kinase (p38 MAPK) -mitogen and stress response kinase 1 (MSK1) signaling cascade, to license TRIM28 to support IFN-I activation (41). Future studies will aim to examine the potential role of TRIM28 S473 phosphorylation and PKR-p38-MSK1 signaling in regulating TRIM28 K63-linked polyubiquitin chain formation.

In line with our findings that TRIM28 plays an essential role in regulating the IFN-I response in both RNA and DNA pathways, which utilize separate PRRs and distinct signaling adaptors, our data further suggests TRIM28 operates via TBK1, a shared downstream component of both pathways. Indeed, TRIM28 associated with TBK1 at the endogenous level, and MEF cells lacking TRIM28 displayed impaired TBK1 phosphorylation upon RNA or DNA pathway stimulation. TBK1 is also known to be subjected to inhibition via the protein phosphatase Mg^2+^/Mn^2+^ dependent 1A (PPM1A), and the E3 ligase TRIM18 has been reported to stabilize PPM1A via K63-linked ubiquitination and aid the recruitment of PPM1A to TBK1 (42–44). Future studies will be necessary to determine the kinetics and mechanisms of TBK1 interactions with TRIM28 and TRIM18/PPM1A to better understand how these E3 ligases fine-tune TBK1 signaling events.

Similar to the importance of TBK1 phosphorylation, several groups have observed TBK1 K63-linked ubiquitination necessitates signaling to IRF3 to elicit IFN-I induction (17). In gain of function experiments, the E3 ligase RNF41 (aka Nrdp1) was shown to promote K63-linked ubiquitination of TBK1 to activate the IFN-I response, while simultaneously targeting the Toll-like receptor (TLR) PRR signaling adaptor, myeloid differentiation primary response gene 88 (MyD88) for K48-linked destructive ubiquitination (45). Additional studies revealed Nrdp1 K63-linked ubiquitination of TBK1 was restricted to lipopolysaccharide (LPS) dependent activation of TBK1 via the TLR4 PRR and was dispensable in the RNA or DNA sensing PRR pathways (45, 46). Alternatively, mind bomb 1 and 2 (MIB1/2) E3 ligases were shown to promote TBK1 K63-linked ubiquitination specifically in the RNA pathway but not in the DNA pathway or TLR pathway (47, 48). So far, the only E3 ligase demonstrated to confer K63-linked ubiquitination of TBK1 downstream of both the RNA and DNA pathway (as well as the TLR4 PRR pathway) is RNF128. However, macrophages lacking RNF128 displayed only a modest reduction in TBK1 K63-linked ubiquitination, TBK1 phosphorylation, and downstream IRF3 phosphorylation and IFN-I induction upon RNA or DNA pathway activation (46). Nevertheless, it may be possible that RNF128 cooperates with TRIM28 to regulate TBK1 activation and will be explored in future studies.

TRIM proteins have classically been described as antiviral restriction factors, often functioning via multiple mechanisms to antagonize viral replication (28, 31, 32). For example, TRIM5α inhibits retroviral infection by targeting the viral capsid as well as by utilizing its E3 ubiquitin ligase function to trigger pro-inflammatory responses via activation of the NF-κB transcription factor (32, 49). Likewise, pioneering experiments identified TRIM28 to suppress endogenous retrovirus transcription events, while later studies additionally found TRIM28 to block HIV-1 integration and silence herpesvirus lytic gene expression (21, 22, 50, 51). Our data connecting TRIM28 to IFN-I activation further uncovers a previously undefined mechanism by which TRIM28 supports antiviral host defenses and ultimately provides new insight into TBK1 regulation in innate immunity (52).

## Materials and methods

### Cell culture, reagents, and antibodies

Murine Embryonic Fibroblast (MEF) cell lines were kindly provided by Dr. Genhong Cheng (University of California, Los Angeles). U937 human monocytes and Human Embryonic Kidney (HEK) 293T cells were obtained from American Type Culture Collection (ATCC). Cells were cultured in Dulbecco’s Modified Eagle Medium (DMEM) (Corning) (MEF and HEK 293T) or Roswell Park Memorial Institute 1640 (RPMI 1640) (Corning) (U937) supplemented with fetal bovine serum (Gibco) (10%) and penicillin/streptomycin (Gibco) (1%) in 5% CO_2_ at 37°C. Poly (I:C) (HMW) and 2’3’-cGAMP were purchased from Invivogen. Calf-thymus DNA and oligos for ISD generation were synthesized by Invitrogen. Recombinant murine IFN-β was purchased from MedChemExpress. An IFN-β enzyme linked immunosorbent assay (ELISA) kit were from PBL Assay Science. Anti-Flag M2 antibody was purchased from Sigma-Aldrich. Other primary antibodies used in this study were GFP, HA, Myc, VSV-G, HSV-1 ICP4 (Santa Cruz Biotechnology); TBK1, p-TBK1, IRF3, p-IRF3, STAT1, p-STAT1 (Cell Signaling Technology); TRIM28, β-Tubulin, GAPDH, RSAD2, control IgG (Proteintech); and K63 ubiquitin (Millipore). Secondary anti-rabbit, anti-mouse, or anti-rabbit Trueblot antibodies conjugated to HRP were purchased from Southern Biotech and Rockland Immunochemicals, respectively.

### Plasmids, cloning, and CRISPR gene editing

Vectors encoding FLAG-TBK1, HA-IRF3, HA-WT, K63, and K48 ubiquitin were generously provided by Dr. Genhong Cheng (University of California, Los Angeles). HA-TRIM28, GFP-TRIM28, and pDONR223-TBK1 were obtained from Addgene. FLAG-TRIM28 was purchased from GenScript. Myc-TBK1 was assembled by Gateway *in vitro* recombination (Thermo Scientific). GFP-TRIM28 C65/68A was generated via site-directed mutagenesis (New England Biolabs) and TRIM28 domain deletions were constructed via PCR using FLAG-TRIM28. To generate TRIM28 KO cells, single guide RNA (sgRNA) targeting murine or human TRIM28 was cloned into pLenticrisprV2 and co-transfected with psPAX2 and pMD2.G packaging and envelope plasmids (Addgene) into HEK 293T cells to produce lentiviral particles to infect MEF or U937 cells, respectively, in the presence of polybrene. Alternatively, human TRIM28 sgRNAs cloned into pLenticrisprV2 were transfected into HEK 293T cells to generate TRIM28 KO HEK 293T cells. Knockout clones were established by puromycin selection for 10 days followed by subsequent limiting dilutions. sgRNA sequences were as follows: murine TRIM28 sgRNA1 GGTACGAACTCCACAGGTCC; murine TRIM28 sgRNA2 GTGCTACTCCAAAGACATCG; human TRIM28 sgRNA GCAGCGGGTGAAGTACACCA; non-targeting control sgRNA CTGAAAAAGGAAGGAGTTGA.

### Transfections, viral infections, and imaging

HEK 293T, MEF cells, and U937 monocytes were seeded in tissue culture dishes/plates 16-18 h prior to polyethylenimine (Polysciences, Inc.) or lipofectamine 2000 (Invitrogen) transfection. VSV expressing GFP (VSV-GFP) and HSV-1 have been described elsewhere (30, 53). MEF cells infected with VSV-GFP were subjected to live imaging using the Echo Revolve fluorescent microscope (kindly supported by Dr. Shitao Li, Tulane University School of Medicine).

### Immunoblot analysis and immunoprecipitation

For immunoblot analysis, cells were harvested in ice cold NP-40 lysis buffer (50 mM Tris-Cl pH 7.4, 150 mM NaCl, 1 mM EDTA, 1% NP-40) supplemented with complete EDTA-free protease inhibitors (MedChemExpress) as described previously (53). Protein concentrations were determined via BCA protein assay (Thermo Scientific). For immunoprecipitation experiments, equal amounts of pre-cleared lysates from transfected cells were incubated overnight with anti-FLAG M2, Myc, TBK1, or IgG control antibodies at 4°C followed by the addition of protein A magnetic beads (MedChemExpress) for 4 hours at 4°C. Captured protein complexes were washed three times with NP-40 lysis buffer containing 250 mM NaCl and then eluted with 2X Laemmli sample buffer containing β-mercaptoethanol. For TBK1 ubiquitination experiments, immunoprecipitated TBK1 was washed three times in radioimmunoprecipitation (RIPA) buffer (50 mM Tris-Cl pH 7.4, 150 mM NaCl, 1% NP-40, 0.5% deoxycholate, 0.1% SDS) containing 10 mM N-ethylmaleimide, followed by an additional RIPA buffer wash containing 1 M urea. All samples were boiled at 95°C for 5 minutes followed by SDS PAGE and immunoblotting onto PVDF membranes. Proteins were detected via enhanced chemiluminescence (Thermo Scientific) using the Amersham Imager 600 (GE Healthcare Life Sciences).

### RNA isolation and quantitative PCR

RNA was isolated using TRIzol reagent (Invitrogen) and converted to cDNA using qScript (Quantabio). Quantitative PCR (Q-PCR) was performed using PerfeCTa SYBR green (Quantabio) in a CFX96 thermocycler (Bio-Rad). Transcript abundance was first normalized to that of mRNA encoding the ribosomal protein L32 or 36B4, then normalized against values for unstimulated controls calculated via the 2^-ΔΔCt^ method. Murine primer sequences were as follows: Ifnb Fwd AGCTCCAAGAAAGGACGAACAT, Rev GCCCTGTAGGTGAGGTTGATCT; Cxcl10 Fwd CCAGTGAGAATGAGGGCCATA, Rev TCGTGGCAATGATCTCAACAC; Ccl5 Fwd GCCCACGTCAAGGAGTATTTCTA, Rev ACACACTTGGCGGTTCCTTC; Mx1 Fwd GACCATAGGGGTCTTGACCAA, Rev AGACTTGCTCTTTCTGAAAAGCC; Ifit1 Fwd ACAGCAACCATGGGAGAGAATGCTG, Rev ACGTAGGCCAGGAGGTTGTGCAT; RPL32 Fwd AAGCGAAACTGGCGGAAAC, Rev TAACCGATGTTGGGCATCAG. Human primer sequences were as follows: IFNB1 Fwd TGTGGCAATTGAATGGGAGGCTTGA, Rev CGGCGTCCTCCTTCTGGAACTG; RPLP0/36B4 Fwd TCGAACACCTGCTGGATGAC, Rev CCACGCTGCTGAACATGCT.

### Statistical analysis

Quantitative data are expressed as mean -fold increase ± S.E. relative to control levels from a representative experiment performed 2-3 times. Statistical significance was determined using ANOVA or student’s t-test (****P<0.0001, ***P<0.001, **P<0.01, and *P<0.05).

## Data availability statement

The raw data supporting the conclusions of this article will be made available by the authors, without undue reservation.

## Ethics statement

Ethical approval was not required for the studies on humans in accordance with the local legislation and institutional requirements because only commercially available established cell lines were used. Ethical approval was not required for the studies on animals in accordance with the local legislation and institutional requirements because only commercially available established cell lines were used.

## Author contributions

FH: Data curation, Formal analysis, Investigation, Methodology, Validation, Writing – review & editing. TN: Investigation, Writing – review & editing. KP: Conceptualization, Funding acquisition, Supervision, Writing – original draft.
